# Kounis syndrome induced by oral intake of aspirin: case report and literature review

**DOI:** 10.11604/pamj.2018.30.301.14948

**Published:** 2018-08-30

**Authors:** Abdelkader Jalil El Hangouche, Ouiame Lamliki, Latifa Oukerraj, Taoufiq Dakka, Nawal Doghmi, Jamila Zarzur, Mohammed Cherti

**Affiliations:** 1 Department of Cardiology B, Ibn Sina Hospital, Mohammed V University, Rabat, Morocco; 2 Exercise Physiology and Autonomic Nervous System Team “EPE-SNA”, Faculty of Medicine and Pharmacy of Rabat, Mohammed V University, Rabat, Morocco; 3 Laboratory of Physiology, Faculty of Medicine and Pharmacy of Tangier, Abdelmalek Essaâdi University, Tangier, Morocco

**Keywords:** Kounis, zavras syndrome, samter´s triad, myocardial infarction, aspirin allergy, coronary spasm

## Abstract

The Kounis-Zavras syndrome is defined as the coincidental occurrence of acute coronary events and hypersensitivity reactions following an allergic reaction including a mast-cell degranulation of vasospastic mediators. This report describes a case of Kounis-Zavras syndrome in the setting of aspirin-induced asthma also known as Samter-Beer triad combining nasal polyps, asthma, and aspirin allergy leading to vasospasm and myocardial infarction. All physicians should be aware of The Kounis syndrome and always keep that unique clinical entity in mind to recognize it promptly and direct the therapy at suppressing the allergic reaction.

## Introduction

Kounis syndrome is currently the term used for allergic angina pectoris or allergic myocardial infarction involving release of inflammatory cytokines through mast cell activation, which leads to coronary artery vasospasm and/or atheromatous plaque erosion or rupture [1, 2]. This syndrome includes the whole clinical spectrum of acute myocardial ischemia, from angina pectoris, the more frequent clinical presentation, to acute myocardial infarction (MI), which is relatively rare [3]. There are three variants of Kounis syndrome have been defined. Several causes have been described ables to induce this syndrome such as drugs, insect stings, foods, environmental exposures and medical conditions [2]. We report a case of Kounis-Zavras syndrome caused by taking aspirin in a woman With Samter's Triad.

## Patient and observation

**Figure 1:**
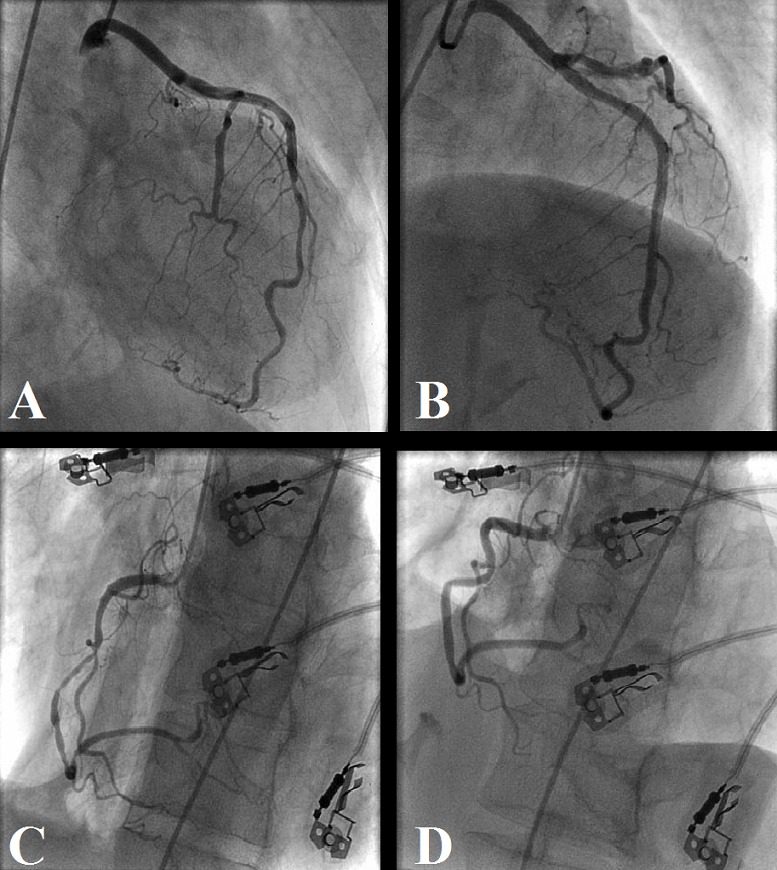
Coronary angiograms before and after intracoronary nitroglycerin; (A) left coronary angiogram with a tubular severe stenosis in the distal LAD and a subtotal occlusion of the first diagonal; (B) left coronary angiogram after intracoronary nitroglycerin injection; the spasm in the distal LAD is completely relieved, the subtotal occlusion of the diagonal is partially improved; (C) right coronary artery (RCA) with patent stent in the proximal segment and diffuse severe stenosis in the mid to distal vessel; (D) RCA after intracoronary nitroglycerin injection with complete relief of the spasm

**Figure 2:**
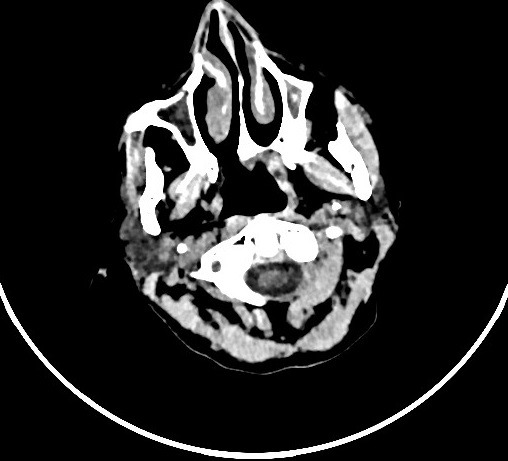
Sinus tomography revealing the presence of bilateral nasal polyposis

A 49-year-old woman with no past medical history or cardiovascular risk factors presents to the emergency department with severe chest pain and shortness of breath. Review of system revealed left hip pain for which patient took aspirin 1 g couple of hours prior to her current presentation. Further history reveals that she experiences similar symptoms of chest pain and shorness of breath after taking aspirin or nonsteroidal anti-inflammatory drugs (NSAIDs). On arrival, the heart rate was 100 bpm, blood pressure: 120/70 mmHg. Physical exam reveals bilateral expiratory lung wheezing. Chest X ray was unremarkable, ECG showed ST segment elevation in the inferior leads. Transthoracic echocardiography showed a kinetic abnormalities of anterolateral and inferior walls with a left ventricule ejection fraction of 35%. The initial troponin I us was makeredly increased with concentration of 50 ng/ml (147 times the normal laboratory value). Coronary angiogram revealed a severe stenosis in the ostium of Right coronary artery (RCA) and a discrete stenosis in the distal left anterior descending artery (LAD). A percutaneous angioplasty was performed on RCA lesion. Repeated echocardiogram showed an improvement of the left ventricule ejection fraction to 50%. Patient was discharged on standard medical therapy including aspirin and clopidogrel. In the subsequent month, patient had multiple recurrences of chest pain asthma attacks and was admitted with a NSTEMI a month later. Repeat coronary angiography showed a patent LAD stent and severe diffuse stenosis affecting all coronary arteries. This time patient was treated with intracoronary nitroglycerin with relieve of symptoms (Figure 1). The new occurrence of asthma attacks associated with asprin consumption was finalty related to Samter's syndrome. Sinus tomography and a nasal endoscopy revealed the presence of bilateral nasal polyposis confirming the diagnosis of the aspirin-exacerbated respiratory disease (AERD) diagnosed as the coexistence of asthma, nasal polyps and aspirin allergy (Figure 2). Aspirin was discontinued and patient discharged on Clopidogrel. Patient remained free of asthma attack and chest pain during two year follow-up.

## Discussion

The association of nasal polyps, asthma and hypersensitivity to aspirin was first described by Widal et al in 1922 and thereafter popularized by Samter and Beers in 1968 referring to this clinical triad [4]. This syndrome has been termed “Syndrome de Widal” or “Samter's Triad.” and it affects 5-10% of patients with asthma. In patients with cardiovascular diseases the incidence is unknown [5]. The pathophysiology of Samter's triad is not fully understood. It's believed that hypersensitivity to aspirin is non-IgE-mediated and results of shunting of the arachidonic pathway by cyclooxygenase-1 inhibition with overproduction of cysteinyl leukotrienes and release of inflammatory mediators, such as tryptase and histamine, from mast cells and eosinophils. It was observed that in these patients cysteinyl leukotrienes and their receptors are elevated at baseline, which considerably increase after NSAIDs ingestion. Since the chronic airway inflammation persists even with strict avoidance of aspirin and other NSAIDs, the ingestion of aspirin simply exacerbates the asthma but does not cause the asthma [2]. The real incidence of the Kounis Zavras syndrome is not well known and most of the data in the literature comes from isolated clinical cases (almost 300) [1-6]. Three variants have been described. Type I variant refers to patients with normal or near normal coronary arteries without predisposing factors for coronary artery disease, in whom the acute allergic attacks can induce coronary artery spasm leading to either coronary spasm without raised cardiac enzymes and troponins or acute myocardial infarction [7, 8]. Type II variant refers to patients with quiescent preexisting atheromatous coronary plaque susceptible to erosion or rupture, while an acute allergic insult, leading to acute myocardial infarction [9]. Type III variant includes patients with late or very late pharmacoactive stent thrombosis. It's thought, in those patients, the hypersensitivity to the compound of nickel alloys, eluted drugs and polymers present in the stent trigger an inflammatory reaction developing over a few days or weeks after the implementation of the stent [6]. This hypothesis is supported by some cases of acute pharmacoactive stent thrombosis occuring in predisposed patients with history of atopy, asthma or other related conditions [7, 10, 11]. Our case had a combined variant of Kounis syndrome (Type I and Type II). Several chemical substances seems to be implicated in Kounis syndrome (tryptase, chymase, histamine) that are massively released in cardiac tissue, coronary arteries and plaques by mast cells during immunological reaction [1-12]. Histamine is the most important one, and has strong vasoconstrictive effects mediated by H1- receptors [7]. Also mast cells are found extensively in and around thrombi, and could contribute to the destabilization and maturation of thrombi by the anticoagulant effect of heparin and tryptase-induced degradation of fibrinogen [13].

Many etiologies of Kounis syndrome have been reported as drugs (antibiotic, analgesic, nonsteroidal antiinflammatory, anticoagulant, corticosteroid, heparin, Intravenous anesthetic, skin disinfectant, Thrombolytic ..) or environmental exposure (ant sting, bee sting, viper venom, wasp sting, jellyfish sting, latex contact, Shellfish eating) [2]. To our knowledge, there is a few cases, reported in the literature, of kounis syndrome occurred in the setting of allergy to acetylsalicylic acid (aspirin), this is the fourth case [2-14]. The sharp rise in cysteinyl leukotrienes after aspirin ingestion in Samter's triad is responsible of myocardial ischemia in patients with normal coronary artery, presumably due to coronary vasospasm [15]. Management of Kounis syndrome consists in treating both allergic and cardiac symptoms, in the same time, which may have contradictory effects; indeed, some administered drugs may aggravate the heart function or worsen the allergic reaction [16]. Guidelines for the treatment of allergic angina pectoris are lacking; therefore, the efficacy of the treatment is based on individuals cases report [1]. In patients with the type I variant, treatment of the allergic event alone may abolish symptoms by using intravenous hydrocortisone and antihistamines. The administration of vasodilators like calcium channel blockers and nitrates may be considered to reduce hypersensitivity-induced vasospasm. In patients with the type II variant, treatment should be started with an acute coronary event protocol, associated with corticosteroids and antihistamines. Whereas, in patients with the type III variant an acute myocardial infarction protocol should be realized with urgent aspiration of intra-stent thrombus [17,18]. However, administration of the usual dose of epinephrine (the drug of choice used in anaphylaxis) in patients under the effect of beta-blockers may exaggerate coronary spasm due to unopposed activity of alpha-adrenergic receptors [17, 19]. Furthermore, beta blockers may enhance the generation and release of anaphylaxis mediators and their effects on end organs [20]. Treatment of aspirin-exacerbated respiratory disease includes the substitution of another category of antiplatelet agents, such as thienopyridines, or a desensibilization as an alternative for patients who are allergic to aspirin and need long-term therapy of aspirin for cardiovascular diseases [5]. We tried a desensibilisation for our patient without much success. Considering the persistence of symptoms and especially considering the frequency, and the risk of recurrence of angina pectoris if spaced taking aspirin; The aspirin intake was stopped and since then, the patient has taken only clopidogrel as antiplatelet agent.

## Conclusion

In recent years, our understanding of Kounis syndrome has improved. In this article, we are reporting a rare clinical entity of coronary spasm caused by taking non-steroidal anti-inflammatory resulting in acute myocardial infarction, and in which case the only eviction of the allergen has led to the cessation of symptoms.

## Competing interests

The authors declare no competing interest.

## References

[1] Fassio F, Losappio L, Antolin-Amerigo D, Peveri S, Pala G, Preziosi D (2016). Kounis syndrome: a concise review with focus on management. Eur J Intern Med.

[2] Schwartz BG, Daulat S, Kuiper J (2011). The Kounis-Zavras syndrome with the Samter-Beer triad. Proc (Bayl Univ Med Cent).

[3] Fassio F, Almerigogna F (2012). Kounis syndrome (allergic acute coronary syndrome): different views in allergologic and cardiologic literature. Intern Emerg Med.

[4] Graefe H, Roebke C, Schäfer D, Meyer JE (2012). Aspirin Sensitivity and Chronic Rhinosinusitis with Polyps: a fatal combination. J Allergy (Cairo).

[5] Christou A, Kafkas N, Marinakos A, Katsanos S, Papanikitas K, Patsilinakos S (2011). Rapid desensitisation of patients with aspirin allergy who undergo coronary angioplasty. Hellenic J Cardiol.

[6] Velasco E, Díaz E, Avanzas P, Rubín JM (2014). Acute stent thrombosis due to Kounis syndrome. International Journal of Cardiology.

[7] Sueda S, Sasaki Y, Habara H, Kohno H (2015). Editorial: Kounis syndrome (allergic angina and allergic myocardial infarction) for cardiologists. Journal of Cardiology Cases.

[8] Gunes H, Turan Sonmez F, Saritas A, Koksal Y (2017). Kounis Syndrome induced by oral intake of diclofenac potassium. Iran J Allergy Asthma Immunol.

[9] Nikolaidis LA, Kounis NG, Gradman AH (2002). Allergic angina and allergic myocardial infarction: a new twist on an old syndrome. Can J Cardiol.

[10] Chen JP, Hou D, Pendyala L, Goudevenos JA, Kounis NG (2009). Drug-eluting stent thrombosis: the Kounis hypersensitivity-associated acute coronary syndrome revisited. JACC Cardiovasc Interv.

[11] Kounis NG, Mazarakis A, Almpanis G, Gkouias K, Kounis GN, Tsigkas G (2014). The more allergens an atopic patient is exposed to, the easier and quicker anaphylactic shock and Kounis syndrome appear: clinical and therapeutic paradoxes. J Nat Sci Biol Med.

[12] Campo G, Pavasini R, Pollina A, Petrini L, Ferrari R (2013). Kounis-Zavras syndrome presenting with ventricular arrhythmias and cardiogenic shock. Journal of Cardiology Cases.

[13] Biteker M (2010). Current understanding of Kounis syndrome. Expert Rev Clin Immunol.

[14] Rayner-Hartley E, Chou A, Saw J, Sedlak T (2016). A Case of Kounis Type I in a Young Woman With Samter's Triad. Can J Cardiol.

[15] Szczeklik A, Nizankowska E, Mastalerz L, Bochenek G (2002). Myocardial ischemia possibly mediated by cysteinyl leukotrienes. J Allergy Clin Immunol.

[16] Cevik C, Nugent K, Shome GP, Kounis NG (2010). Treatment of Kounis syndrome. Int J Cardiol.

[17] Kounis NG (2013). Coronary hypersensitivity disorder: the Kounis syndrome. Clin Ther.

[18] El Hangouche AJ, Alaika O, Doghmi N (2017). Kounis-Zavras Syndrome: from Pathogenesis to Management. Journal of Cardiology & Cardiovascular Therapy.

[19] Ioannidis TI, Mazarakis A, Notaras SP, Karpeta MZ, Tsintoni AC, Kounis GN (2007). Hymenoptera sting-induced Kounis syndrome: effects of aspirin and beta-blocker administration. Int J Cardiol.

[20] Kounis GN, Hahalis G, Kounis NG (2008). Anaphylaxis, beta blockade and the Kounis syndrome. Paediatr Anaesth.

