# Corrigendum: Kounis syndrome induced by oral intake of aspirin: case report and literature review

**DOI:** 10.11604/pamj.2019.33.172.19430

**Published:** 2019-07-04

**Authors:** Abdelkader Jalil El Hangouche, Ouiame Lamliki, Latifa Oukerraj, Taoufiq Dakka, Nawal Doghmi, Jamila Zarzur, Mohammed Cherti

**Affiliations:** 1 Department of Cardiology B, Ibn Sina Hospital, Mohammed V University, Rabat, Morocco; 2 Exercise Physiology and Autonomic Nervous System Team "EPE-SNA", Faculty of Medicine and Pharmacy of Rabat, Mohammed V University, Rabat, Morocco; 3 Laboratory of Physiology, Faculty of Medicine and Pharmacy of Tangier, Abdelmalek Essaâdi University, Tangier, Morocco

**Keywords:** Corrigendum, Kounis, zavras syndrome, samter´s triad, myocardial infarction, aspirin allergy, coronary spasm

## Abstract

This corrigendum corrects article "Kounis syndrome induced by oral intake of aspirin: case report and literature review" and its publication references ie The Pan African Medical Journal. 2018;30:301. doi:10.11604/pamj.2018.30.301.14948.

## Corrigendum

The original version of this article [1] had an author name reversed in the article citation on Pubmed. The name of the author has been corrected by inverting author's last name and the first name as follows; "Hangouche AJE" to "El Hangouche AJ".
